# Adaptive Localization of Focus Point Regions via Random Patch Probabilistic Density from Whole-Slide, Ki-67-Stained Brain Tumor Tissue

**DOI:** 10.1155/2015/673658

**Published:** 2015-02-22

**Authors:** Yazan M. Alomari, Siti Norul Huda Sheikh Abdullah, Reena Rahayu MdZin, Khairuddin Omar

**Affiliations:** ^1^Pattern Recognition Research Group, Center for Artificial Intelligence Technology, Faculty of Information Science and Technology, Universiti Kebangsaan Malaysia, 43600 Bangi, Malaysia; ^2^Department of Pathology, UKM Medical Center, Universiti Kebangsaan Malaysia, 56000 Cheras, Kuala Lumpur, Malaysia

## Abstract

Analysis of whole-slide tissue for digital pathology images has been clinically approved to provide a second opinion to pathologists. Localization of focus points from Ki-67-stained histopathology whole-slide tissue microscopic images is considered the first step in the process of proliferation rate estimation. Pathologists use eye pooling or eagle-view techniques to localize the highly stained cell-concentrated regions from the whole slide under microscope, which is called focus-point regions. This procedure leads to a high variety of interpersonal observations and time consuming, tedious work and causes inaccurate findings. The localization of focus-point regions can be addressed as a clustering problem. This paper aims to automate the localization of focus-point regions from whole-slide images using the random patch probabilistic density method. Unlike other clustering methods, random patch probabilistic density method can adaptively localize focus-point regions without predetermining the number of clusters. The proposed method was compared with the *k*-means and fuzzy *c*-means clustering methods. Our proposed method achieves a good performance, when the results were evaluated by three expert pathologists. The proposed method achieves an average false-positive rate of 0.84% for the focus-point region localization error. Moreover, regarding RPPD used to localize tissue from whole-slide images, 228 whole-slide images have been tested; 97.3% localization accuracy was achieved.

## 1. Introduction

Cancer is a leading cause of death worldwide. In Malaysia, more than 30,000 deaths from cancer have been reported annually with most of these cases being diagnosed at an advanced stage [1].

The analysis of microscopy images is extremely important in both the medical and computer science fields. Analysis of whole-slide tissue images is an important part of cancer diagnosis. However, manually selected tissue slide focus point regions do not capture the complete information available to pathologists during initial microscopic analysis.

The diagnostic evaluation of a patient with suspected cancer typically comprises several steps, including a brain scan, often magnetic resonance imaging (MRI), as a first step. If imaging increases the suspicion of a brain tumor, a brain biopsy is usually performed. A biopsy is a procedure that involves the removal of a small portion from the tumor area so that the cells or tissues can be examined [2]. This sample is next treated and sliced in a pathology laboratory, and then the histological structure of the tissue cells is examined under the microscope by a pathologist. For diagnostic purposes, usually each diagnostic process involves staining the specimen with specific dyes [3].

**Figure 1:**
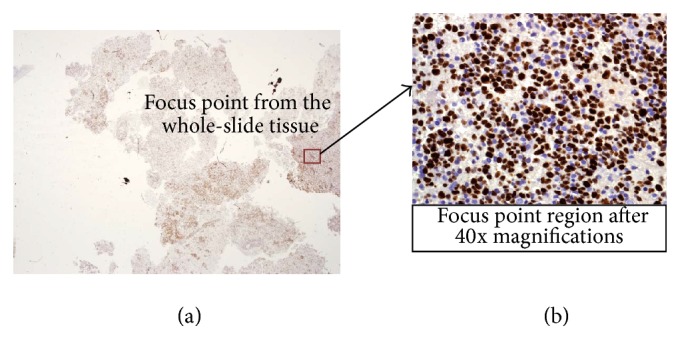
(a) Whole-slide tissue image sample for Ki-67-stained histology image for a brain tumor. The red box represents a sample for the focus point from the whole-slide tissue. (b) Image captured after 40x magnification for the focus point region.

Regarding performing PRE, pathologists usually stain the tissue using Ki-67 antigen [4]. After staining the tissue, the pathologists then examine this biopsy tissue to make a diagnosis. This process starts with visualization of the stained tissue using a whole slide under the microscope at low magnifications (1–1.5x). From the whole slide, the focus point regions that are highly concentrated in cancerous cells (stained cells) are identified and localized as shown in Figure 1. Next, for each selected focus point region, the pathologist creates a zoom region until 40x magnification to perform further analysis for these parts. PRE is then carried out for each part.

Two strategies for selection of the focus point region to differentiate the tumor area from the whole-slide tissue: in the first strategy, pathologists select the regions that exhibit the highest stained cell concentration; that is, tumor heterogeneity is examined by localizing the tissue areas with a high density of positively stained cells that are also known as focus point or hot spot regions [5, 6]. In the second strategy, pathologists select the regions systematically sampled to cover the whole tissue area [7]. However, manual focus point region selection from the tissue slide does not extract all available information in the initial microscopic analysis [8]. In addition, the process of identifying the focus point regions from the whole-slide tissue is highly subjective, using eye pooling or eagle-view techniques, with high variation of interpersonal and intrapersonal observations and lack of reproducibility [9]. The accuracy of PRE mainly depends on the number of focus points localized from the whole slide and selection of the correct focus point regions from the slide. An increase in the number of focus point regions indicates that the pathologist covered most of the whole-slide tissue area, leading to an increase in the accuracy of PRE and an effect on the patient treatment plan [10].

The problem of localizing the focus point regions from the whole-slide tissue image can be addressed as a clustering problem in image processing [9, 11–13]. Our objective in this paper is to propose an adaptive localization method for Ki-67 staining of whole-slide tissue for histology images of a brain tumor. The purpose of this localization method is to identify all of the focus point regions from the whole-slide tissue images in bounding boxes and calculate the maximum number of focus point regions that will help all pathologists regardless of the strategies they followed. This approach may help pathologists, particularly junior pathologists, to identify tumor regions that exhibit high proliferating activity, called “focus point regions,” and will support them as a second opinion in their analysis during the PRE process. Qualitative analysis of the whole slide to identify the focus point regions under the microscope is extremely tedious, prone to errors, time-consuming particularly when numerous slides need to be diagnosed in practice, and subject to interpersonal and intrapersonal observation. Recent studies have shown that the intrapersonal observation variations can be up to 20% [14]. Thus, automation of this process can increase the pathologist's precision, reduce interpersonal observation, save time, and provide support as a second opinion to help in the diagnosis and analysis via introduction of quantitative analysis. The proposed method was evaluated by three pathologists; the false positive rate was 0.84% with a localization accuracy of 99.1%. In the following sections of this paper, we will summarize work related to whole-slide analysis (Section 2), present the methodology used (Section 3), discuss the results and experiments (Section 4), and review the conclusions.

## 2. Related Work

Using clustering and segmentation methods highly depends on the application, imaging modalities, and other factors. Each image modality has its own characteristics to be extracted to perform the segmentation. Therefore, there is no single segmentation method with acceptable results for all medical imaging modalities [9]. According to that challenge, medical imaging segmentation remains a problem for this field [11]. There are different approaches in medical image segmentation with some approaches based on heuristics, region growing, edge detection, and thresholding methods [15]. Other approaches use machine learning techniques, including supervised, unsupervised, and semisupervised techniques [11, 16], whereas other approaches need prior information in the form of atlases [17].

Many researchers have investigated whole-slide tissue analysis using unsupervised techniques. Nadernejad and Sharifzadeh [18] presented a new pixon-based method for image segmentation. They form a pixonal image using a bilateral filter, which is used as a kernel function. Some advantages of using this filter include decreased image noise, help in smoothing the image, prevention of over segmentation problems, and removal of unwanted noises from the environment. In the next step, the fuzzy *c*-means model is used to segment the obtained pixonal image. Their experimental results showed less computational and better accuracy than other segmentation techniques.

Xinwu [19] presented some modifications in the original *k*-means clustering algorithm. He claimed the original *k*-means has some limitations such as low efficiency in the way that *k*-means performed the centroid calculations, affecting the *k*-means efficiency. Therefore, a new method was proposed to overcome this limitation. Additional preprocessing steps were added to the input images to speed up and enhance the clustering process. Next, he improved the clustering seed method through movement of the seed clustering toward intensive data areas. In addition, the original *k*-means was stuck in the local maximum solution in some cases. Solving this problem was achieved through using the proposed method as a local searching process to inlay in the local search structure of the iteration. The proposed method outperforms the original *k*-means method through the local search and extends the searching range. A comparison with original *k*-means using 3D medical volume images was conducted; his proposed method achieved 94.7% accuracy, while the original *k*-means accuracy was 77.5%. Another important limitation in the *k*-means not discussed in this paper is predetermining the number of clusters.

Some researchers use unsupervised learning methods to segment and localize the tissue from the whole-slide images. Hiary et al. [11] presented a method to segment the tissue from the whole-slide image and localize it in a bounding box using a *k*-means algorithm. They aimed to extract only the regions of interest, which were represented in the tissue and were bounded in the box, before entering the slide into the slide scanner. This step saves both the scanning time and memory space required. The unsupervised clustering *k*-means method achieved 96% accuracy. Using unsupervised clustering methods still needs to predetermine the number clustering but does not need a ground truth in their case, unlike their previous work [16] when they used a supervised machine learning method to perform the same task of segmentation and localization. They made a hybrid method to combine heuristic methods with parametric machine learning. Color, intensity, texture, and spatial distribution features were used in the segmentation and localization of the tissue. In addition to using principal component analysis in feature reduction, subsequent training in two layers in back propagation neural networks was required. The accuracy achieved was 96% with ground truth and data training required.

Hybridization in clustering methods was performed as in [9], and Lopez et al. presented a hybrid method to localize hot spots in the whole-slide tissue for ki-67-stained histology images. Hot spot regions in the whole slide help identify the tumor regions that exhibit high proliferating activity. They proposed a clustering method that could localize an unknown number of clusters. This method was carried out using proposed hybridization between hierarchical clustering (HC) and nonhierarchical clustering techniques. They proposed a hybrid method combining the DBSCAN algorithm and standard single linkage (HC) method. The DBSCAN algorithm was used to initiate the single linkage method by specific instances instead of starting from the individual data. They applied their method on Ki-67-stained whole-slide pathology images and compared the proposed method results with the manual hot spots labeled by the pathologist. The results showed some improvement in consistency between the pathologists regarding the hot spot tasks and support the pathologists regarding quantitative descriptors. The resulting clusters from the hybrid clustering method used in this paper can be highly variable in terms of shape, size, and density. Unfortunately, variability in cluster size may confuse pathologists, when creating higher magnifications of this region for further analysis. Therefore, if all hot spots clusters bounded by a box have the same size for all boxes and were found in all regions suggested to be visited by the pathologist, it will be easier for the pathologists to visit all of the boxes for further analysis if needed. In addition, this method is highly sensitive to parameters, which is hard to determine for all cases.

Elie et al. [7] presented a simple method for quantifying the focus point regions that represents stained cells from the whole-slide tissue. Two thresholding steps were used: first, the tissue was extracted; second, the stained cell regions from the tissue were extracted. Thereafter, a morphology close operator is used to combine all of the neighbored pixels. Next, reconstruction of size 10 is performed using the open morphology operator to keep only the large stained areas. Finally, AND logical operator is applied between a manual marked mask image and the binary image, stained pixels and focus point regions. One drawback of this technique is that some parameters are not easy to determine and are not easy to set as a fixed value suitable for all cases, such as thresholding, because they used fixed thresholding in certain steps. Thus, these values can significantly influence the results.

## 3. Methodology

Current clustering algorithms such as *k*-means and fuzzy *c*-means can handle this challenge. However, they still have limitations of the computation time needed and preknowledge of the number of clusters. These limitations are solved in our technique. Therefore, a new technique for the Ki-67-stained histology images for the whole-slide tissue aims to cluster and localize all focus point regions (FPRs). This technique clusters all regions that are highly concentrated in the cancerous cells from the tissue. In our case, we need an adaptive clustering technique that can identify all clusters from the tissue without preknowledge about the number of clusters. Therefore, we propose the random patch probabilistic density (RPPD) method to identify all focus point regions from the tissue. This method outperforms *k*-means and fuzzy *c*-means clustering methods in the processing time required to find all clusters. In addition, unlike *k*-means and fuzzy *c*-means clustering methods, RPPD adaptively finds all required clusters without predetermining the number of clusters.

**Figure 2:**
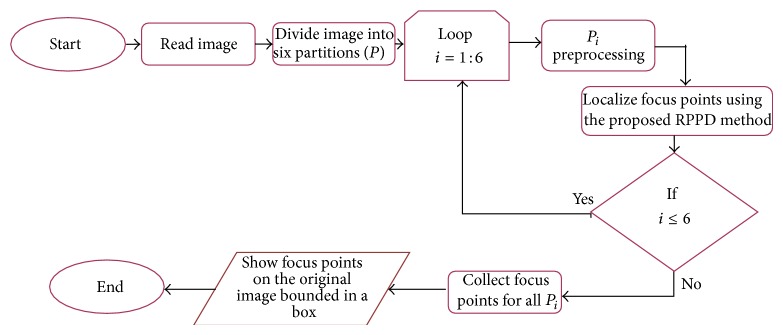
General methodology for the focus point localization proposed method.

In this focus point regions localization problem, three general steps are performed as shown in Figure 2. The first step aims to divide the whole-slide tissue image into six partitions. This division was carried out based on the size of the image (4140 × 3096). In addition, some images have uneven illumination due to their capture by camera; therefore, shadow problems appeared due to the direction of illumination. Thus, a single threshold will not be effective. The partitioning step enhanced the thresholding results, which affected the step of extracting tissue from the whole-slide image using the adaptive Otsu thresholding method. Another advantage of the partitioning step is to localize the focus point regions locally from all parts in the image. After division, each partition is subsequently considered a separated input image. The second step is a global image segmentation technique using the Otsu thresholding method for each image partition. This step aims to extract the tissue from the whole-slide image. The third step is to follow a local structural segmentation approach on the extracted tissue only by using the RPPD proposed method. This step aims to identify a particular structure in the tissue. This structure in our case refers to the highly concentrated cell regions; that is, high density cell regions from the tissue.

### 3.1. Preprocessing

The proposed RPPD method for focus point region localization works with binary images: microscopy images of Ki-67 whole-slide tissue are colored images. These images are captured by a digital camera from the microscope. Therefore, in such images, some brightness problems appeared while capturing the images. Thus, in this phase, whole-slide RGB images follow some preprocessing steps to solve the problem of brightness and to covert the image to gray and then to a binary image. These steps are as follows.

- The RGB partition *P*_*i*_ image is converted to a gray-scale image by eliminating the hue and saturation information while retaining its luminance.
- Histogram equalization: this step uses a contrast-limited adaptive histogram equalization (CLAHE) method to transform the values of the gray-scale image to enhance the contrast of the image. The (CLAHE) method works locally in the image instead of the whole image and is carried out by dividing the image into small regions called tiles. Next, histogram equalization is applied for each tile to enhance the contrast, and then the results are included in the whole image.
- The gray partition *P*_*i*_ image is converted into a binary image using the Otsu thresholding method to extract the tissue from the whole-slide image. The Otsu method chooses the threshold to minimize the intraclass variance of the black and white pixels [20]. These segmentation phases were applied for all partitions in each image.

### 3.2. Other Localization Methods

In the proposed RPPD method, localization of the tissue was performed based on the density feature in each box. However, existing studies [4, 11, 19, 21] usually employ *k*-means and fuzzy *c*-means to select the region of interest or localize a specific object in an image. In these partitioning clustering approaches, each cluster is represented by its center, which may not be a part of the dataset. Additionally, the number of clusters is fixed, and each object assigned to the nearest cluster center is based on a distance measure. The latter fact usually causes incorrectly cut-off borders between clusters. Furthermore, all clusters have approximately the same size. *k*-means and fuzzy *c*-means algorithms are the most known centroid-based methods. They work by randomly selecting the initial clusters and then assigning each object to the nearest cluster. These methods have many drawbacks. The final results are highly dependent on the initial clusters chosen, the methods are highly sensitive to outliers, failure of localization often occurs, and the number of clusters must be specified in advance [22, 23].

When comparing with other clustering methods such as *k*-means [22, 24],

(1) $$\begin{array}{c} J=\overset{k}{\underset{j-1}{\sum }}\;\overset{n}{\underset{i-1}{\sum }}{\left\|x_{i}^{j}-c_{j}\right\|}^{2}, \end{array}$$

where ‖*x*_*i*_^*j*^ − *c*_*j*_‖^2^ is the chosen distance measure between a data point *x*_*i*_^*j*^ and the cluster center *c*_*j*_ is an indicator of the distance of *n* data points from their respective cluster centers.

When comparing with the fuzzy *c*-means clustering method [25]

(2) $$\begin{array}{c} J_{m}=\overset{N}{\underset{i-1}{\sum }}\;\overset{C}{\underset{j-1}{\sum }}U_{ij}^{m}{\left\|x_{i}-C_{j}\right\|}^{2},\;1\leq m\leq \infty , \end{array}$$

where *m* is any real number greater than 1, *U*_*ij*_ is the degree of membership of *x*_*i*_ in the cluster *j*, *x*_*i*_ is the *i*th of *d*-dimensional measured data, *C*_*j*_ is the *d*-dimension center of the cluster, and ‖∗‖ is any norm expressing the similarity between any measured data and the center.

The same preprocessing steps were performed to extract the tissue. The binary-extracted tissue pixels, which represent the dark staining regions of the tissue, are clustered using *k*-means [24] and fuzzy *c*-means [25] based on the tissue pixel coordinates. The clustering methods clustered the tissue pixels based on distances between pixels, indicating that the tissue pixels are close together in a cluster. In other words, clustering the tissue is performed based on the tissue pixel concentration or density, similar to that based on RPPD.

### 3.3. The Proposed Random Patch Probabilistic Density (RPPD)

In general, segmentation in images is defined by the regions to be identified as in (3), FPR_*k*_ ∈ Ω, where Ω is the segmented tissue and needs to be segmented into regions FPR_*k*_ and

(3) $$\begin{array}{c} \overset{N}{\underset{k}{⋃}}\text{FP}{\text{R}}_{k}=Ω-\left(\sim \text{FPR}\right),\;0\leq N\leq \left(m\times n\right), \end{array}$$

where *N* is the number of regions and *R*_*j*_∩*R*_*k*_ = *ϕ*, ∀*j* ≠ *k*, (*m* × *n*) is the image size. However, good image segmentation softens the condition of a hard subset to only one region by assigning probabilities of pixels to lie in regions [16].

The objective of the RPPD is to detect the highly concentrated cell regions (focus point regions; FPRs) by minimizing false positive (FP) focus point regions.

*Terminology Definition I*

*P*_*i*_: image partition,
Ω: array of pixels for the segmented tissue in *P*_*i*_,
Ω^*t*^: number of tissue pixels (black pixels) inside the candidate box,
${Ω}^{\overset{ˇ}{t}}$: number of nontissue pixels (white pixels) inside the candidate box,
*F*_*i*_: random pixel selected from Ω,
CFP: candidate focus point region,
*D*_*i*_: density feature of the tissue inside candidate CFP,
FPR: CFP becomes a true focus point region if it meets the *D*_*i*_ feature,
*T*_min⁡_: the minimum number of pixels remaining in Ω to continue searching for more focus points,
*T*_*r*_: the threshold value represents the accepted ratio to consider CFP as FPR.

**Figure 3:**
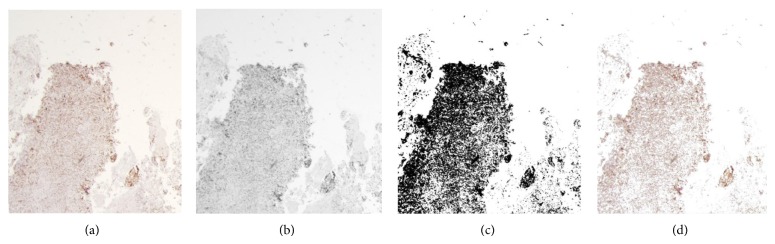
(a) Original tissue image, (b) gray-scale tissue image, (c) segmented binary tissue after Otsu thresholding, and (d) segmented tissue after returning to the original colors from the original image for the corresponding pixels.

After segmenting each partition *P*_*i*_ using the Otsu thresholding method, the segmented image has only dark region-stained tissue, which is needed to identify FPR in Ki-67 histology-stained images. This segmented tissue represents the concentrated regions of the tissue.  Figure 3(a) illustrates the original Ki-67-stained image, and Figure 3(d) illustrates the segmented tissue after thresholding. Therefore, in the next step, the proposed method should cluster this tissue to regions based on its density features and high cell-concentrated regions, similar to what pathologists do.

#### 3.3.1. The Basic Idea for the Proposed RPPD

**Figure 4:**
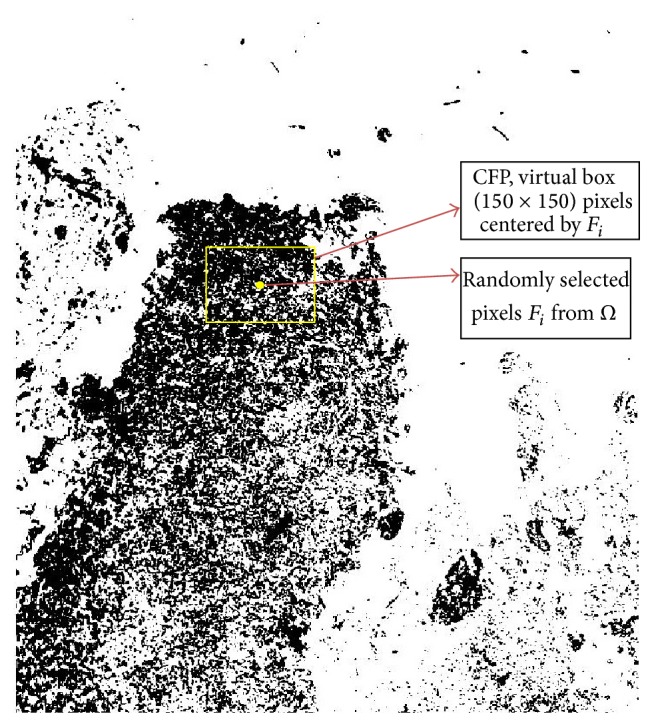
Randomly selected point from tissue and CFP.

Our proposed RPPD method involves binary images. The black pixels represent the tissue; therefore, all of the tissue pixels are stored in tissue array Ω. A pixel, *F*_*i*_, is chosen randomly from Ω. A virtual box is then drawn with a size of 150 × 150 pixels, and *F*_*i*_ is set to be the center of this box. This box is considered a candidate focus point region (CFP). In the next step, this CFP is checked based on density feature criteria to decide whether it will be considered a CFP.  Figure 4 shows the randomly selected *F*_*i*_ and the virtual box centered by *F*_*i*_. Using this random patch localization, the main limitation for the current clustering method, which is the preknowledge concerning the number of clusters, can be solved. In addition, all tissue parts are checked locally from the image.

Many experiments were performed to determine the choice of box size. The trade-off between larger and smaller box size is the number of boxes at the end. Each true box represents a focus point region. When the size of the box is small, the number of focus point regions will be large in the outcome. If the size of the box is large, the number of focus point regions will be small in the outcome. In our case, the size of the images was huge. Thus, we used a medium-sized box (150 × 150 pixels) to obtain an outcome with a reasonable number of focus point regions that can be conveniently applied for the two strategies for focus point region selection by pathologists.

#### 3.3.2. Determining Candidate Focus Point Regions

In the next step, RPPD identifies the density feature of the tissue inside the box based on (4) as shown in Figure 4. RPPD accepts or rejects this region based on the formula shown in (5). If the value of *D*_*i*_ is greater than a threshold value *T*_*r*_, then CFP is considered a true FPR. Next, RPPD removes all pixels inside the box from Ω and moves on to select a new *F*_*i*_. Otherwise, if the value of *D*_*i*_ is less than a threshold value *T*_*r*_, RPPD rejects this CFP and restarts by selecting a new *F*_*i*_. With the assumption of a 150 × 150 box size, CFP is summarized as follows:

(4) $$\begin{array}{c} D_{i}=\frac{{Ω}^{t}}{{{Ω}^{t}+Ω}^{\overset{ˇ}{t}}}, \end{array}$$

(5) $$\begin{array}{c} \sum \text{CFP}=\left\{\begin{array}{cc} \text{Reject} & D_{i}<T_{r}\\ \text{Accept} & D_{i}\geq T_{r}\\ \text{Remove}\;\;{Ω}^{t}\;\;\text{from}\;\;Ω & D_{i}\leq 10\%. \end{array}\right. \end{array}$$

**Figure 5:**
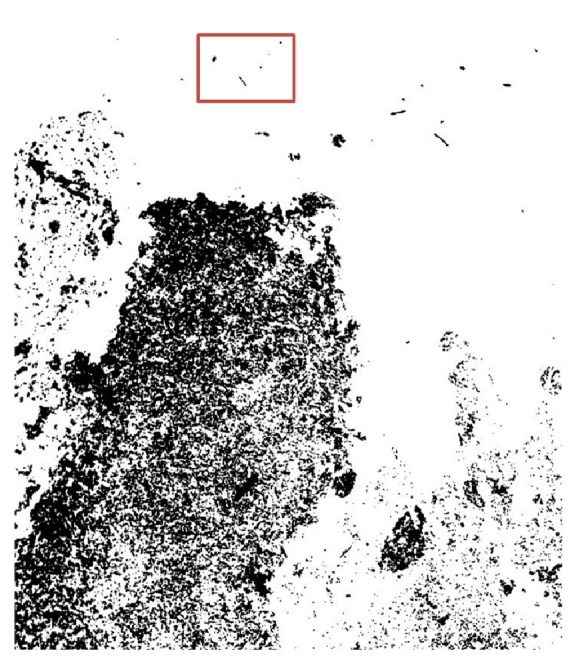
Insignificant regions of tissue found and ignored by RPPD.

If the density inside the box is less than 10%, RPPD removes the pixels inside this box. In some cases, during the slide preparation process, some small pieces of tissue spread through the slide. These tissue pieces are not significant regions for the pathologists. Therefore, RPPD ignores such pieces of tissue as shown in Figure 5.

#### 3.3.3. RPPD Iteration Number

RPPD continues searching for focus point regions in the image *P*_*i*_ until it meets one of two conditions:

- the number of pixels in Ω is less than *T*_min⁡_;
- the number of iterations exceeds 100 times without identifying any FPR.

The value of *T*_min⁡_ is technically defined by the maximum number of tissue pixels inside the box required to keep this CFP region rejected from RPPD to consider a FPR. It can be formulated as

(6) $$\begin{array}{c} T_{\min}=\left[\frac{{Ω}^{t}}{{{Ω}^{t}+Ω}^{\overset{ˇ}{t}}}<15\%\right]. \end{array}$$

This number of remaining tissue pixels in Ω is not sufficient to decide a FPR even if these pixels are found in a candidate box. Therefore, if the number of remaining tissue pixels has reached *T*_min⁡_, the RPPD proposed method terminates the iterations.

Conversely, a number of iterations exceeding 100 without identifying any FPR indicate that most of the FPRs are found, and the probability of finding a new FPR is very weak in the partition image *P*_*i*_. This value of 100 is determined based on experimental tests; moreover, the partition image size is not large.

#### 3.3.4. Our Proposed Scaling to Accept CFP as a Focus Point Region (FPR)

In this step, if the *D*_*i*_ value inside the box is greater than a threshold value *T*_*r*_, then the RPPD method deems this CFP a FPR. RPPD has criteria to accept CFP as a FPR based on the formula shown in (7). This criterion is dependent on a multiscale of the threshold value *T*_*r*_. The value of the threshold *T*_*r*_ is used in the RPPD proposed method to represent different scales of priority, with each scale having a different box color displayed in the resulting image. The following categories are supposed to provide pathologists with an indication of the FPR found:

(7) ∑FPR=Red  Box      Di≥75%High  priorityBlue  Box     50%≥Di≥75%Middle  priorityGreen  Box 30%≥Di≥50%Low  priorityBlack  Box   15%≥Di≥30%Lowest  priority.

In the results, each category of focus point regions has a different box color; this step gives the pathologists a detailed indication about the FPR found and helps them to choose the FPR process.

#### 3.3.5. The Proposed RPPD Steps

**Figure 6:**
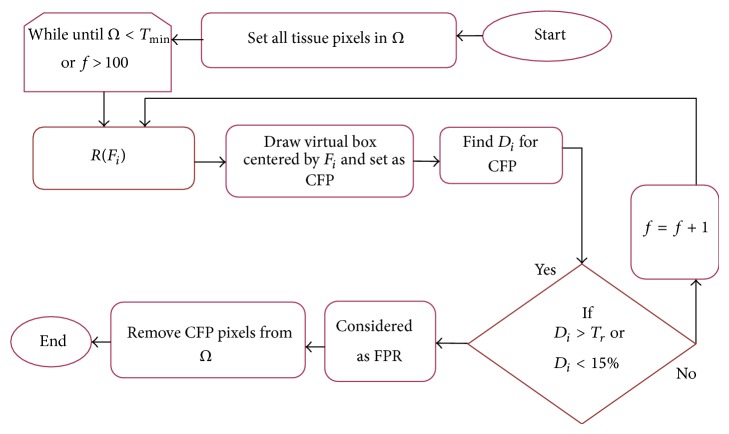
The proposed RPPD method workflow.

The workflow of the proposed RPPD method for each *P*_*i*_ is shown in Figure 6.

**Step 1 .**

The entire binary image is divided into six small partition images. Each partition is denoted by *P*_*i*_ so that each image is defined by the set *P*_1_, *P*_2_,…, *P*_*i*_, where *P* is the partition image and *i* is the number of partitions.

**Step 2 .**

For each *P*_*i*_, all extracted tissue pixels are stored in Ω_*i*_. *f* is defined as the number of failures that can be tolerated. The failure counter is initialized so that *f* is set to 0. $T_{\min}={Ω}^{t}/{{(Ω}^{t}+Ω}^{\overset{ˇ}{t}})<15\%$ is defined and set, and then the value of *T*_*r*_ is defined and set.

**Step 3 .**

The algorithm loops from *P*_1_ to *P*_*i*_.

**Step 4 .**

When |Ω_*i*_ | >*T*_min⁡_ or *f* ⩽ 100, where *f* is the number of failures that can be tolerated, and *T*_min⁡_ is the maximum number of tissue pixels inside the box required to keep this CFP region rejected from RPPD to be considered a FPR, the algorithm proceeds to Step 5; otherwise it goes to the next *P*_*i*_.

**Step 5 .**

A random pixel *F*_*i*_ is selected from Ω_*i*_, and then a virtual box is drawn, and *F*_*i*_ is placed in the center. This box region is defined as a candidate focus point (CFP).

**Step 6 .**

If number of tissue pixels inside this CFP meets the *D*_*i*_ feature, RPPD considers this region a FPR. All of the tissue pixels from Ω_*i*_ are then removed, and a new *F*_*i*_ is selected. Otherwise, if *D*_*i*_ is less than 15%, all of the tissue pixels from Ω_*i*_ are also removed and *f* = *f* + 1 is performed, and a new *F*_*i*_ is selected. Otherwise, *f* = *f* + 1 is performed and the algorithm goes to Step 5.

In conclusion, the proposed RPPD aims to detect and localize all focus points from whole-slide Ki-67-stained tissue. This problem can be solved using the clustering methods described in the literature [9].

## 4. Experimental Results and Discussion

### 4.1. Dataset

#### 4.1.1. Self-Collected Dataset to Localize Focus Point Regions from the Whole Slide

Our dataset contains thirty images of whole-slide tissue of Ki-67-stained histology images. The images represent brain tumor cases that include diffuse large B-cell lymphoma, atypical meningioma grade II, rhabdoid meningioma grade III, atypical choroid plexus papilloma grade II, and anaplastic astrocytoma grade III. We used these images to localize focus point regions that pathologists focused on to create zoom regions and conduct further analysis to perform PRE. The images are from the Hospital University Kebangsaan Malaysia. The histologic images in the dataset were captured using an Olympus BX50 microscope (Olympus Corporation, Japan). All of the images were captured using a DP72 digital camera (Olympus Corporation) and cellSens Life Science imaging software, version 1.6 (Olympus Corporation). All of the images are in the tiff format with a resolution of 4140 × 3096. The images were taken at 1x magnification.

#### 4.1.2. Dataset for Bounding the Tissue from the Whole Slide to Be Scanned

**Figure 7:**
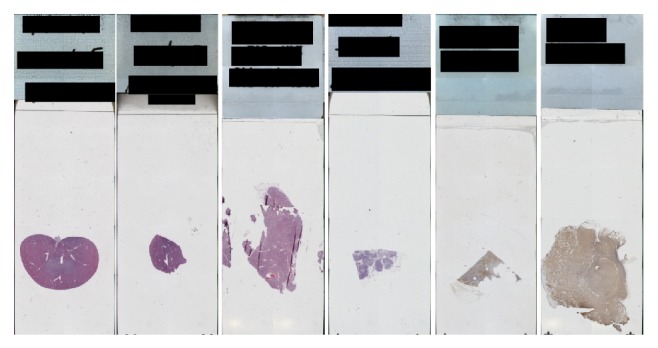
Sample of the whole-slide tissue dataset to be scanned.

In this dataset, 228 images used for the whole-tissue slide which were used in [11, 16] varied in color, size, shape, and location in the slide. In this dataset, our proposed method was used to localize the tissue from the whole-slide image and fix a box around the tissue. The required memory and time to scan the whole slide were very large. The purpose of this step was to determine approximately the area to be scanned by the digital pathology scanner from the whole slide. The scanner will only scan the area inside the box. This area must include the localized tissue from the whole slide. This method will save scanning time and memory space required.  Figure 7 shows some sample images from this dataset.

### 4.2. Evaluation Methods

#### 4.2.1. Self-Collected Dataset

Focus point region localization from the whole-slide tissue for Ki-67 histology images is a very challenging task because inter- and intrapersonal observation are very high among pathologists. In medical image analysis, a major concern is interpersonal observation reliability. Experience, environment, data, and human factors all contribute to the expert decision variability in the medical domain. Therefore, it is very difficult to find a gold standard for this problem. Thus, in this paper, three senior pathologists checked the proposed method results independently. All of them agreed in the identification of the false positive focus point regions; however, in true-positive and false negative cases, they have interpersonal observations. False positive regions refer to the regions identified by the proposed method as incorrect regions, such as regions outside the tissue borders. True-positive regions refer to the regions identified by the proposed method as correct regions. Moreover, false negative focus point regions refer to correct focus point regions that are not detected by the proposed method. The agreement between pathologists in determining the same true-positive focus point regions is low. They find the focus point regions in a very subjective manner based on their experience. Additionally, in some cases, the interpersonal observation had reached 20% [14].

Moreover, in some cases, pathologists examine the whole-slide tissue focusing on highly concentrated cancerous cells; at the same time, they may also focus on nonconcentrated regions.

For these reasons, it is very difficult to find a gold standard for this problem. Therefore, in this paper, the evaluation method used focused on identifying the false positive rate based on pathologist evaluation.

We found the false positive rate by identifying number of incorrect boxes. The area for each incorrect box was determined, and then the total area for all these boxes was calculated. Thereafter, total area of the incorrect boxes was divided by the area of the whole image based on

(8) False  Positive  Rate  =box  size∗number  of  incorrect  boxesimage  size×100.

The false positive rate was calculated for each image, and then the dataset was divided into five sets. For each set, we determined the average false positive rate (AFPR), which occurs when agreement exists between the pathologists and proposed method for the incorrect focus point region.  Table 1 shows the average false positive rate for each set of images with the focus point region localization accuracy for each set. Furthermore, the total average false positive rate and focus point region localization accuracy was determined for all of the image sets.

#### 4.2.2. Dataset for Bounding the Tissue from the Whole Slide to Be Scanned

In this dataset, our proposed method was used to localize the tissue from the whole slide and fix a box that contains the localized tissue. For the evaluation, if the box localizes all of the tissue, it was considered true. If the box missed a significant area of the tissue, it was considered a localization error, and the image was counted as an error (localization error). The localization accuracy was calculated as in

(9) $$\begin{array}{c} \text{Accuracy}=\left(1-\frac{L_{\text{Error}}}{N}\right)100\%. \end{array}$$

The box size used in the proposed method was (40 × 40) pixels, and the *T*_*r*_ threshold value used was 10%. After identifying all of the boxes in the image, the maximum box that contains all the small boxes in the image was then found. This step was performed to localize the tissue in one box.

For the 228 whole-slide tissue images, the localization accuracy was 97.3% using our proposed method. Our proposed method outperformed the method used in [11, 16], which used unsupervised and supervised learning methods to localize the tissue from the whole slide and then bounded the tissue image in a box.

**Figure 8:**
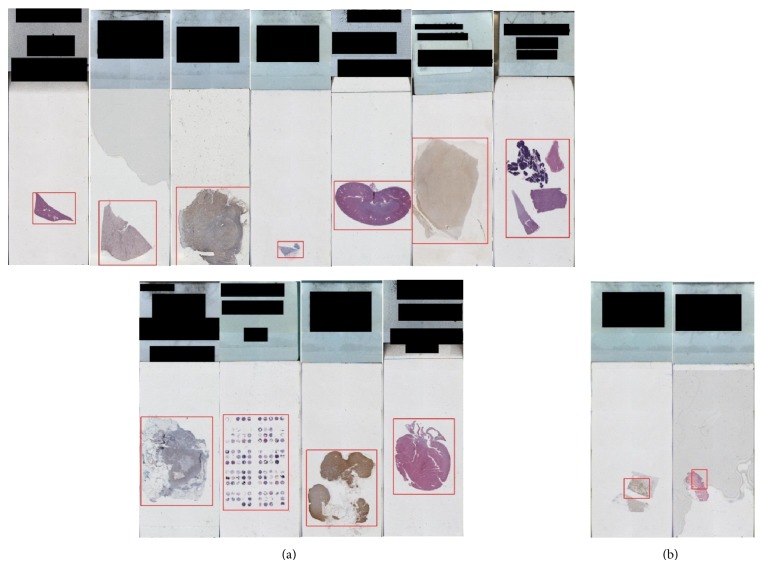
(a) Sample correct tissue localization results and (b) sample incorrect tissue localization results for the proposed RPPD method.

The RPPD proposed method could localize tissue using different sizes, shapes, and colors.  Figure 8 shows some sample results for the RPPD proposed method using tissue localization from the whole slide; (a) shows some correct tissue localization, and (b) shows some incorrect tissue localization.

### 4.3. Discussion

**Figure 9:**
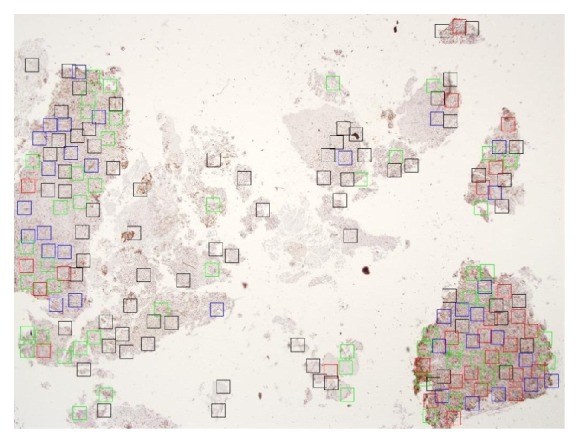
Sample of focus point regions localized by the RPPD proposed method with multiscaling results. The red boxes indicate high priority. The blue boxes indicate middle priority. The green boxes indicate low priority. The black boxes indicate the lowest priority.

In clinical routines, pathologists use their experience to localize some focus point regions from whole-slide tissue. They then further analyze the selected regions after zooming in to 40x magnification to perform PRE for that case.  Figure 9 shows a sample of focus point regions localized by the RPPD proposed method from the Ki-67 whole-slide tissue image. In such cases, some tissue regions have more cancerous cell concentration, and these regions are used by pathologists objectively for further analysis.

According to interpersonal observation in determining the true-positive and true-negative regions, a priority criterion for the localized focus point regions was suggested. Four focus point region categories are suggested as mentioned in Section 3.3.4 as a box coloring. Each category represents a localized region with specific priority. Using these criteria, the RPPD proposed method could identify most focus-point regions in the whole-slide tissue, even the regions with low significance to some pathologists. Pathologists can then choose some of these focus point regions to complete the analysis. Using this method, the proposed RPPD can reduce interpersonal observation and identify more focus point regions.

Moreover, in medical image analysis, particularly regarding the focus point region localization cases, the major concern is to reduce the false positive rate while identifying the most focus-point regions existing in the tissue. Pathologists do not care to examine all of the focus point regions in the whole slide; they just use some focus point samples to make a decision for that case. In addition, not all pathologists use the same focus point regions to make the decision due to interpersonal observation. These clinical settings of the pathologist's routine motivated us to propose an adaptive localization method that can find most of the focus point regions, even with less significance, and then pathologists can choose from them for further analysis. In addition, this method would reduce the false positive rate, which can waste the pathologist's time and efforts.

**Figure 10:**
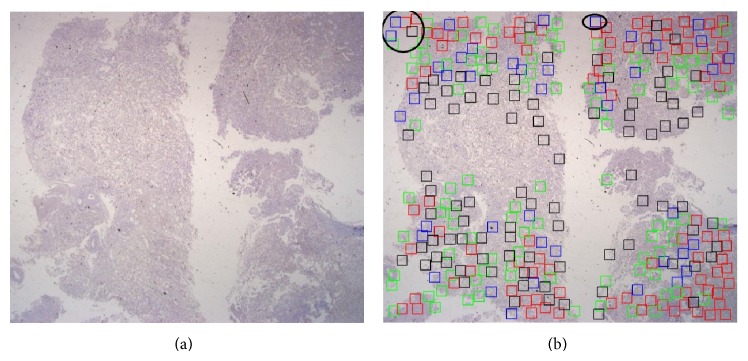
(a) Original whole-slide tissue image, (b) some false positive regions labeled in the black circles after localization using the proposed RPPD method due to brightness and segmentation errors.

In our proposed method, some false positive regions were caused by tissue segmentation errors from the preprocessing steps. Images used in this paper were captured using a digital camera fixed on the microscope; thus, some brightness problems occurred during the image capture that can affect tissue extraction as shown in Figure 10.  Figure 10(a) shows the original image, and Figure 10(b) shows the focus point regions; the box inside the black circles refers to false positive regions.

**Figure 11:**
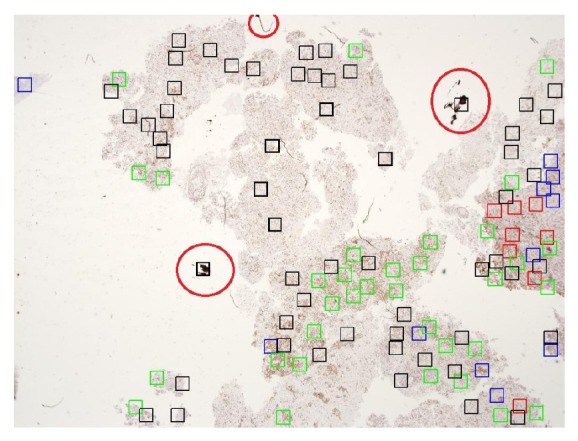
Regions in the red circles have problems in slide preparation such as staining problems.

In some cases, false positive focus point regions arise from problems in preparing the slide by pathologists. In the slide preparation, problems in staining can sometimes cause some noise in the image, affecting the focus-point region localization as shown in Figure 11 as regions inside the red circles.

**Figure 12:**
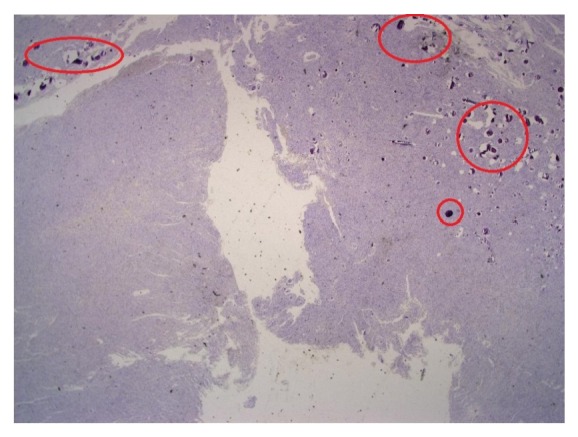
Regions in the red circles have problems in slide preparation such as air bubbles and tissue folds.

In some cases, false positive focus point regions arise from problems in slide preparation by the pathologists. In the slide preparation, problems such as fold or air bubbles sometimes cause noise in the image, affecting the focus-point region localization as shown in Figure 12 as regions inside the red circles.

### 4.4. Comparison of the Results with Other Methods

Focus point region localization is a clustering problem that can be handled by known clustering methods such *k*-means and Fuzzy *c*-means. Thus, we compared our proposed RPPD method with *k*-means and fuzzy *c*-means methods.

We conducted our experiments for the focus-point region localization using both *k*-means and fuzzy *c*-means clustering methods. Two experiments for each method were performed using a different number of clusters. The first experiment was conducted using 120 clusters, while the second experiment used 150 clusters for both *k*-means and fuzzy *c*-means methods as shown in Tables 2 and 3. The false positive rate when using 120 clusters was lower than that when using 150 clusters in both *k*-means and fuzzy *c*-means methods. Unfortunately, even when the false positive rate is low with 120 clusters, the number of true-positive focus point regions found was not comprehensive to cover all of the suspected cases due to interpersonal observations. Therefore, more clusters are needed to be localized. In addition, and for reasonable comparison with the RPPD proposed method, the average focus point regions found in RPPD are 250 per image.

Experimentally, we determined that the number of clusters used should be not very large because of the running time and its effects on the number of false positive clusters: when the number of clusters is larger, the false positive rate is increased.

**Figure 13:**
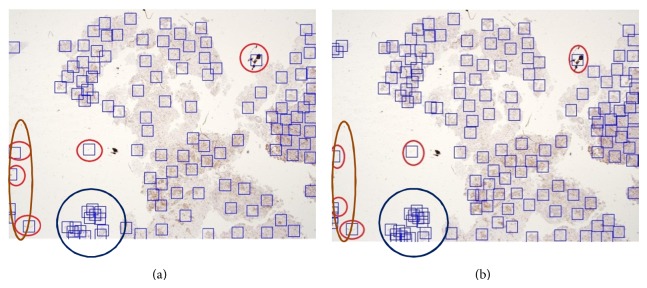
Results of focus point regions using *k*-means with (a) 120 clusters and (b) 150 clusters.

**Figure 14:**
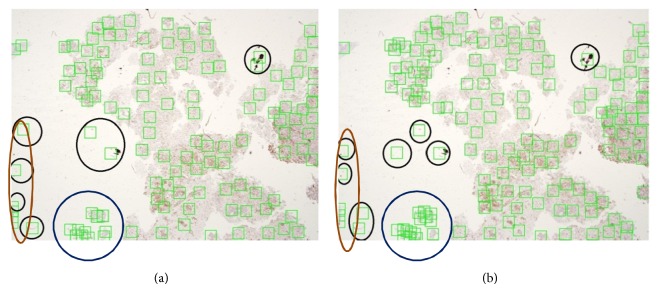
Results of focus point regions using fuzzy *c*-means with (a) 120 clusters and (b) 150 clusters.

Figures 13 and 14 show sample results using *k*-means and fuzzy *c*-means clustering methods with (a) 120 clusters and (b) 150 clusters, respectively. All of the red circles indicate false positive regions, and the blue circles represent the area that, at most, needs no more than two focus point regions. However, a high concentration of overlapping focus point regions is observed using the *k*-means method. Figure 11 shows the same case using our proposed RPPD method with a low false positive rate. Moreover, the regions marked on the brown oval represent areas of tissue that should not be detected because they are not significant to the pathologists, and they are not reasonable areas of tissue to examine. They might represent problems in some slide preparations.

The RPPD proposed method outperforms *k*-means and fuzzy *c*-means in two major issues. RPPD works adaptively without the need to predetermine the number of clusters, which is considered a very difficult task in focus-point region localization. In addition, RPPD has a faster processing time than *k*-means and fuzzy *c*-means.

From a clinical standpoint, results from *k*-means and fuzzy *c*-means have a higher rate of false positive ratio in focus-point region localization, unlike our proposed RPPD method, which has a lower false positive rate.

Table 4 summarizes the comparison among the RPPD proposed method, the *k*-means method and the fuzzy *c*-means method. This comparison includes the average false positive rate and average running time for each method. The different numbers of clusters were tested in *k*-means and fuzzy *c*-means. The first experiment used 120 clusters, whereas the second experiment used 150 clusters. Table 4 shows each experiment for each method, the average false positive rate, and the average run time.

As a conclusion, the proposed RPPD method outperforms both the *k*-means and fuzzy *c*-means clustering methods in focus-point regions localization from the whole-slide tissue stained by Ki-67; the RPPD proposed method achieved less false positive average rate and shorter running time using different number of clusters. In addition, the proposed RPPD method is able to localize all focus-point regions adaptively without predetermining the number of regions to be localized, while in *k*-means and fuzzy *c*-means, the number of regions to be localized must be predetermined, which is considered a very challenging and subjective task. Furthermore, the process of automating the focus-point regions should support the two strategies followed by the pathologists, which helps in reducing the intra/interpersonal observation. Thus, adaptive localization of focus-point regions is more effective in reducing the intra/interpersonal observation than asking the pathologists to predetermine the number of focus-point regions.

## 5. Conclusions

This paper proposed a method that adaptively localizes focus point regions from whole-slide Ki-67-stained histopathology images. The random patch probabilistic density method can localize the tissue based on the density feature of an unknown number of clusters. This method solves two major problems that current clustering methods encounter. The first is that RPPD can cluster data to an unknown number of clusters. The second problem is that the RPPD running time is too short compared with the current method on the same dataset and computer device. The RPPD method was tested on self-collected dataset for whole-slide tissue images and achieved a 0.84% false positive rate. Compared to the *k*-means and fuzzy *c*-means clustering methods with the RPPD method, the results achieved using the *k*-means and Fuzzy *c*-means methods were good, but the number of clusters has to be predetermined, and they had longer running times than our proposed RPPD method. The RPPD method can help pathologists identify focus point regions to proceed in the PRE process. RPPD identifies most focus-point regions in the tissue using simple priority criteria, and these criteria can serve as two strategies that pathologists can follow to localize focus point regions. In addition, our method helps to reduce the interpersonal observation between pathologists. Moreover, RPPD is used to localize the tissue from whole-slide images; it has been tested using 228 whole-slide images, and 97.3% localization accuracy was achieved.

## Figures and Tables

**Table 1:** Summary of results for AVPR.

Set	Average false positive rate	Localization accuracy
1	1.20%	98.7%
2	1.03%	98.9%
3	0.28%	99.7%
4	0.70%	99.3%
5	0.91%	99%

Total	0.82%	99.1%

**Table 2:** Summary of results for the *k*-means method using 120 and 150 clusters.

Set	Average false positive rate (120 clusters)	Average false positive rate (150 clusters)
1	1.02%	1.40%
2	0.63%	0.91%
3	0.34%	0.40%
4	0.76%	0.85%
5	1.52%	1.90%

Total	0.85%	1.09%

**Table 3:** Summary of results for the fuzzy *c*-means method using 120 and 150 clusters.

Set	Average false positive rate (120 clusters)	Average false positive rate (150 clusters)
1	0.94%	1.46%
2	0.85%	0.91%
3	0.31%	0.37%
4	0.67%	0.88%
5	1.43%	1.90%

Total	0.84%	1.10%

**Table 4:** Comparison of the average false positive rate for the RPPD, *k*-means, and fuzzy *c*-means algorithms.

Method	Average false positive rate	Average run time
*k*-means (120 clusters)	0.85%	821.23 seconds
*k*-means (150 clusters)	1.09%	1074.83 seconds
Fuzzy *c*-means (120 clusters)	0.84%	1875.79 seconds
Fuzzy *c*-means (150 clusters)	1.10%	2426.36 seconds
RPPD proposed method	0.84%	239.65 seconds
